# New horizons in adaptive capacity and heat-related health in older people: integrating frailty, energy insecurity and social resources

**DOI:** 10.1093/ageing/afag315

**Published:** 2026-11-01

**Authors:** Laura Petraglia, Maddalena Conte, Valentina Parisi, Grazia Daniela Femminella, Dario Leosco

**Affiliations:** Department of Translational Medical Sciences, University of Naples Federico II, Via Sergio Pansini, 5, Naples, NA 80131, Italy; Department of Translational Medical Sciences, University of Naples Federico II, Via Sergio Pansini, 5, Naples, NA 80131, Italy; Department of Translational Medical Sciences, University of Naples Federico II, Via Sergio Pansini, 5, Naples, NA 80131, Italy; Department of Translational Medical Sciences, University of Naples Federico II, Via Sergio Pansini, 5, Naples, NA 80131, Italy; Department of Translational Medical Sciences, University of Naples Federico II, Via Sergio Pansini, 5, Naples, NA 80131, Italy

**Keywords:** heatwaves, older people, adaptive capacity, energy insecurity, social frailty

## Abstract

Extreme heat is a major environmental threat to healthy ageing, with older people experiencing the highest rates of heat-related morbidity and mortality. Although age-related physiological changes, multimorbidity and frailty remain key determinants of heat susceptibility, climate change, social inequalities and energy insecurity are reshaping the conditions in which older people experience and respond to thermal stress. This narrative New Horizons review integrates evidence identified through a structured, non-systematic search of peer-reviewed literature and relevant institutional reports across geriatric medicine, environmental health and climate science. It examines how frailty, intrinsic capacity, housing, energy affordability and social frailty influence the ability of older people to recognise risk, access protection and respond effectively to extreme heat. Particular attention is given to energy insecurity and summer cooling poverty, through which limited access to affordable household energy may undermine resilience. Ageing models primarily characterise individual vulnerability and functioning within the environment, whereas climate vulnerability models organise risk around exposure, sensitivity and adaptation; we use adaptive capacity to connect these perspectives by asking whether personal and contextual resources can be mobilised for protection. It may provide a conceptual basis for developing and subsequently validating a heat-adapted comprehensive geriatric assessment and for informing targeted prevention and climate adaptation policies for ageing populations.

## Key points

- Extreme heat in older adults reflects more than biological susceptibility.
- Frailty, intrinsic capacity and social context jointly shape heat resilience.
- Energy insecurity and cooling poverty may amplify indoor heat exposure and heat-related health risks.
- Comprehensive geriatric assessment could incorporate heat-specific factors that shape adaptive capacity.

## Introduction

Extreme heat is a major environmental threat to public health [1]. Europe, the fastest-warming continent, has warmed at approximately twice the global rate since the 1980s and is experiencing more frequent, longer and more intense heatwaves [2–5]. In summer 2022, an estimated 61 672 heat-related deaths occurred across Europe, approximately three-quarters among adults aged 65 years or older [6].

Population ageing is a global transition. By 2050, nearly one in three Europeans will be aged 65 years or older [7], and more than two-thirds of the world’s population aged 60 years or older will live in low- and middle-income countries. An additional 177–246 million adults aged 69 years or older may experience dangerous acute heat exposure by mid-century, predominantly in Asia and Africa [8]. Climate change and population ageing will therefore increase the burden of heat-related illness across diverse geographical and socioeconomic settings [2].

Older adults’ susceptibility to heat is well established [9–13]. Age-related impairment of thermoregulation, declining physiological reserve, multimorbidity and polypharmacy compromise responses to thermal stress and increase heat-related illness and mortality [1, 9–11, 14, 15].

These biological responses occur within physical, social and institutional environments that shape exposure and access to protection. Housing quality, urban planning, income, gender, rurality, institutionalisation, services and affordable energy create territorial differences in thermal experience and adaptive opportunities [5, 16–21]. Evidence remains concentrated in Europe and other high-income settings, limiting transferability to the Global South [21].

Environmental gerontology and person–environment models interpret outcomes through the interaction between individual competence and environmental demands [22]. Intrinsic capacity describes individual’s physical and mental capacities, functional ability and their interaction with the environment, while resilience looks at the ability to withstand or recover from stressors [23–25]. Adaptive capacity adds a climate-specific dynamic dimension. Individual, social, material and institutional resources can be mobilised to anticipate, cope with and recover from thermal stress [5, 26, 27].

Accordingly, this narrative review aims to integrate geriatric, environmental and climate-related evidence through an adaptive-capacity perspective, examining how frailty and intrinsic capacity interact with housing and social resources, how energy insecurity shapes heat vulnerability and how these domains could inform geriatric assessment and public planning.

## Methodology

This narrative review was informed by structured, non-systematic searches of PubMed/MEDLINE, Web of Science and Scopus from inception to 31 August 2026, combining terms for ageing or older people and extreme heat with frailty, intrinsic or adaptive capacity, resilience, housing, energy insecurity, social vulnerability and health or social care. We considered peer-reviewed studies, reviews and conceptual papers without geographical restriction, supplemented by authoritative institutional reports and citation tracking. Selection prioritised relevance, methodological robustness, geographical diversity and clinical or policy applicability; indirect or inconsistent evidence relevant to the narrative synthesis was included, with its limitations explicitly acknowledged in the text. Evidence was synthesised narratively without formal risk-of-bias assessment. The tables and Figure 1 are author-developed, non-validated outputs.

**Figure 1:**
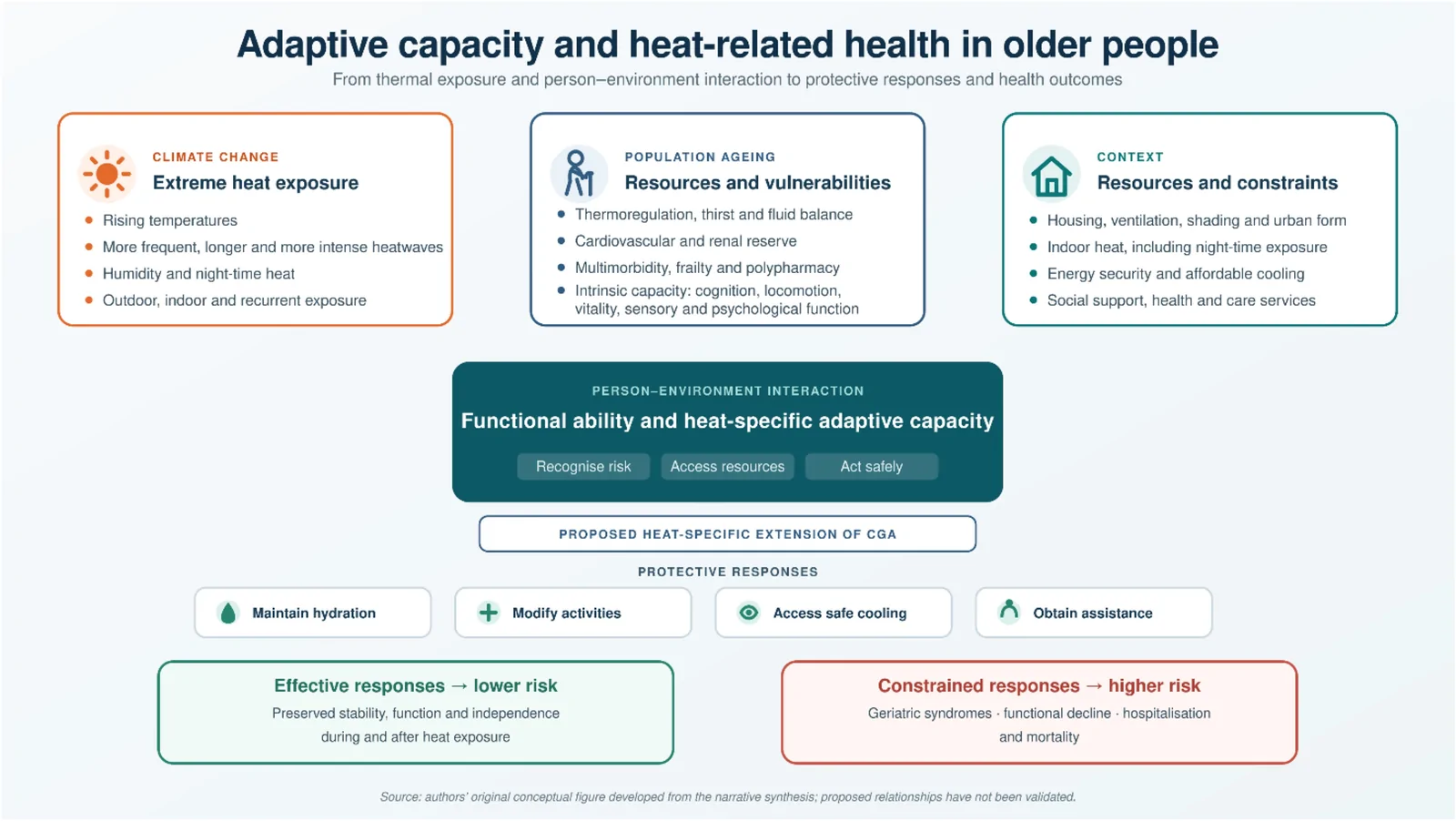
Adaptive capacity linking extreme heat to health outcomes in older people. Extreme heat interacts with individual resources and vulnerabilities, including physiological and clinical reserve, frailty and intrinsic capacity and with contextual resources and constraints, including housing, energy security, social support and health and social care. This person–environment interaction shapes functional ability and heat-specific adaptive capacity, influencing whether effective protective responses can be mobilised. Effective responses may reduce heat-related risk, whereas constrained responses increase the risk of geriatric syndromes, functional decline, hospitalisation and mortality. Source: Authors’ original conceptual figure developed from the narrative synthesis; the proposed relationships have not been validated.

## Biological susceptibility: What physiology explains

The increased susceptibility of older adults to heat-related illness is supported by robust experimental evidence and represents one of the best-characterised pathophysiological paradigms in ageing research. Thermoregulation relies on coordinated cardiovascular, renal, neuroendocrine and behavioural responses that preserve physiological stability during heat exposure. Ageing progressively impairs these mechanisms, reducing heat dissipation and tolerance to thermal stress and increasing the risk of dehydration, organ dysfunction and death [9–11].

Heat dissipation depends primarily on cutaneous vasodilatation and sweating, both of which decline with advancing age. Older adults exhibit reduced sweat production, delayed sudomotor activation and a diminished capacity to increase skin blood flow, resulting in slower heat loss and greater heat storage during prolonged exposure [9, 10, 28–30].

Cardiovascular adaptation is likewise impaired. Maintaining thermal stability requires an increase in cardiac output and redistribution of blood flow towards the skin. Age-related reductions in cardiovascular reserve limit both responses, increasing cardiac workload while reducing heat dissipation, particularly in people with cardiovascular disease [9, 11, 29, 31].

The ageing kidney further contributes to heat vulnerability. Declining glomerular filtration, urinary concentrating capacity and responsiveness to osmotic stimuli impair fluid and electrolyte regulation. Together with reduced thirst perception, these changes increase the risk of dehydration, hypernatraemia and acute kidney injury during prolonged heat exposure [32].

Multimorbidity and commonly prescribed medicines may further compromise cardiovascular, renal and heat-dissipating responses, increasing susceptibility to heat-related complications [11, 15].

Beyond acute decompensation, repeated heat exposure may have cumulative implications for biological ageing. Recurrent dehydration, cardiovascular strain, systemic inflammation, oxidative stress and sleep disruption could progressively erode physiological reserve, although their contribution as mediating pathways remains insufficiently established. In US adults aged 56 years or older, greater neighbourhood heat exposure over periods ranging from days to 6 years was associated with acceleration of several DNA-methylation clocks [33]. A longitudinal study of middle-aged and older Chinese adults linked high temperature combined with low relative humidity to acceleration of an AnthropoAge-derived measure [34]. These findings extend the potential effects of heat beyond acute illness; however, observational designs, outdoor exposure estimates and heterogeneous biomarkers preclude causal inference and leave uncertain whether the changes are transient, cumulative or reversible.

These established age-related changes affect multiple interdependent pathways and substantially reduce tolerance to thermal stress [9–11, 15, 32]. Their clinical consequences often emerge through common geriatric syndromes.

## Extreme heat as a precipitant of common geriatric syndromes

In older people, dehydration, delirium, syncope, falls, medication-related adverse events and functional decline may be interconnected manifestations of thermal stress exceeding available reserve. Presentation may be non-specific: confusion, reduced mobility, a fall or unexplained loss of independence may be the first sign of heat-related illness. Dehydration develops when cutaneous and respiratory fluid losses are not matched by intake; reduced thirst, lower total body water, impaired renal concentrating ability, dysphagia, cognitive impairment, mobility limitation and dependence on others magnify risk. The resulting volume and electrolyte disturbances may precipitate hypernatraemia, acute kidney injury, hypotension, cardiovascular decompensation and delirium. Prevention requires access to fluids and individualised hydration plans, with monitoring of renal function, electrolytes or body weight in selected high-risk patients; heart failure, advanced kidney disease and prescribed fluid restriction require tailored advice rather than generic recommendations [9–11, 15, 32]. Delirium may result from hyperthermia, dehydration, electrolyte disturbance, hypotension, sleep disruption and altered drug clearance, while cognitive impairment limits recognition of risk and protective behaviour. Extreme heat is associated with hospitalisation among people with dementia [35], but this does not establish incident delirium, for which direct epidemiological evidence remains limited.

Volume depletion superimposed on cutaneous vasodilatation may reduce cerebral perfusion and precipitate orthostatic hypotension or syncope, particularly in people with impaired baroreflexes, autonomic dysfunction, cardiovascular disease or blood-pressure-lowering treatment [9, 11, 15, 29, 31]. Falls may occur during presyncope or reflect weakness, fatigue, impaired attention, sleep disruption, reduced postural stability and medication-related sedation. High indoor temperatures have been associated with poorer gait speed and chair-rise performance, although epidemiological evidence quantifying heat-attributable falls remains limited [36]. Polypharmacy can increase vulnerability through fluid and electrolyte loss, impaired sweating, restricted cardiovascular responses, hypotension, sedation and reduced renal drug clearance. Evidence varies across drug classes: a recent systematic review supports effects of anticholinergic agents and non-selective beta-blockers on core temperature, whereas many other warnings rely on indirect or younger-population data [15, 37]. Heat-specific medication review should be anticipatory and individualised, considering indication, dose, renal function and hydration; appropriate medicine storage should also be verified in homes, hospitals and long-term care facilities, as excessive temperatures may compromise the stability of some medicines [38]. Automatic or unsupervised withdrawal should be avoided.

Extreme heat may also impair function through fatigue, muscle weakness, sleep disturbance, reduced intake, disease decompensation and activity restriction. Acute reductions in mobility and self-care may be transient in robust individuals but can initiate more consequential trajectories in those with frailty or limited intrinsic capacity. Dehydration, delirium, syncope or a fall may lead to emergency care or hospitalisation, followed by immobility, deconditioning and loss of independence. Functional deterioration then further restricts access to fluids, cooling and assistance, creating a self-reinforcing cycle. The persistence of functional loss after individual or recurrent heatwaves remains uncertain [36]. Table 1 summarises the principal mechanisms, clinical indicators and preventive or management implications.

**Table 1:** Extreme heat as a precipitant of common geriatric syndromes in older people: Underlying mechanisms, clinical indicators and preventive or management implications.

Syndrome or outcome	Predisposing factors and mechanisms	Clinical indicators	Prevention and management
Dehydration and electrolyte disturbances	Reduced thirst, total body water and renal concentrating ability; increased fluid losses; cognitive or mobility impairment, dysphagia or dependence limiting fluid access; diuretics, laxatives and medicines affecting renal haemodynamics	Reduced intake or urine output; acute weight loss; dry mucosa; postural hypotension; tachycardia; electrolyte abnormalities; declining renal function	Individualised hydration plan and assisted fluid access; monitor weight, renal function and electrolytes in high-risk patients; tailor advice in heart failure, advanced kidney disease or prescribed fluid restriction
Delirium and acute cognitive deterioration	Hyperthermia; dehydration; electrolyte disturbance; hypotension; acute kidney injury; sleep disruption; altered drug clearance; dementia, sensory impairment, frailty or previous delirium	Acute or fluctuating inattention, altered awareness or behaviour; agitation or lethargy	Monitor cognition and behaviour; identify and correct precipitants; provide cooling and appropriate hydration; involve caregivers; consider heat exposure in otherwise unexplained cognitive deterioration
Orthostatic hypotension and syncope	Cutaneous vasodilatation; reduced central blood volume; dehydration; impaired baroreflex or autonomic function; limited cardiovascular reserve; diuretics and vasoactive medicines	Postural dizziness, weakness or visual disturbance; orthostatic blood-pressure reduction; presyncope; transient loss of consciousness; unexplained fall	Assess postural symptoms, blood pressure and hydration; correct volume depletion when appropriate; review diuretic and vasoactive treatment individually; advise gradual postural changes and provide mobility assistance
Falls	Orthostatic hypotension or syncope; weakness and fatigue; impaired attention, gait, transfers or postural stability; sleep disruption; medication-related sedation	New unsteadiness; impaired gait or transfers; near-falls or falls associated with dizziness, confusion or weakness; reduced safety during usual activities	Reassess fall risk; ensure safe access to fluids and cooling; review hypotensive and sedative medicines; provide supervision or mobility support; preserve safe physical activity
Polypharmacy-related adverse events	Diuretic- or laxative-related fluid and electrolyte loss; impaired sweating from anticholinergic medicines; restricted cardiovascular responses with non-selective beta-blockers; vasoactive hypotension; sedative or psychotropic effects; reduced renal drug clearance	Hypotension; electrolyte disturbance; acute kidney injury; confusion or excessive sedation; impaired mobility; reduced intake; new symptoms associated with heat exposure or dehydration	Individualised medication review considering indication, dose, renal function, hydration and therapeutic index; monitor high-risk treatments and verify appropriate medicine storage; provide contingency advice; avoid automatic or unsupervised discontinuation
Acute or persistent functional decline	Fatigue; weakness; sleep disturbance; reduced nutritional or fluid intake and activity; disease decompensation; delirium, syncope or falls; hospitalisation, immobility and deconditioning	Reduced gait speed, transfer ability, endurance or ADL; reduced intake; increased dependence; incomplete recovery after heat-related illness	Assess mobility, ADL and support needs before, during and after heatwaves; preserve safe activity, nutrition and hydration; initiate early mobilisation and rehabilitation; increase caregiver, healthcare or community support as needed

*Source:* Authors’ own elaboration based on the narrative synthesis. ADL, activities of daily living.

## Frailty and intrinsic capacity: Explaining heterogeneity in heat vulnerability

Although physiological mechanisms explain much of the increased susceptibility of older adults to heat, clinical outcomes depend on the interaction between multiple physiological systems, chronic disease, functional status and biological reserve. This multidimensional perspective has shifted geriatric medicine from disease-centred models towards resilience, defined as the capacity of the organism to withstand a stressor and maintain or restore equilibrium [39, 40]. Frailty is the geriatric construct that most directly captures loss of resilience.

The Fried phenotype identifies physical manifestations of frailty, whereas the deficit-accumulation model quantifies the cumulative burden of health deficits [39–42]. Both approaches describe frailty as the consequence of multisystem decline and reduced reserve, distinguishing it from the coexistence of chronic diseases or disability. Even moderate thermal stress may therefore precipitate disproportionate clinical consequences in a frail person. Limited compensatory reserve increases the risk of acute decompensation, hospitalisation and death, making frailty informative beyond chronological age and multimorbidity [9, 11–13].

Recent epidemiological studies support this interpretation, showing that frailty identifies older adults at increased risk of adverse heat-related outcomes independently of chronological age and multimorbidity [12, 13]. Frailty may amplify the consequences of heat through the cumulative reduction of reserve across multiple organ systems.

Multimorbidity and polypharmacy contribute to this multidimensional vulnerability through cumulative effects on mobility, cognition, hydration and functional reserve, in addition to organ-specific physiological effects. Their relevance therefore depends on the individual’s overall clinical and functional context rather than on disease or medicine counts alone [15, 37, 39, 40].

Intrinsic capacity complements frailty by describing the composite of physical and mental capacities on which an individual can draw across the life course [23–25, 43]. It encompasses five interacting domains, locomotion, cognition, vitality, psychological capacity and sensory function and emphasises the resources that remain available to the individual and their evolution over time.

During extreme heat, these domains influence the ability to recognise environmental risk, perceive thirst or thermal discomfort, maintain hydration, modify daily activities, operate cooling devices, reach a cooler environment and seek assistance. These responses require integrated functioning across several domains rather than isolated organ competence. Modest impairments distributed across cognition, mobility, vitality or sensory function may therefore compromise protection even when no single deficit appears severe.

Frailty and intrinsic capacity consequently provide distinct but related perspectives: frailty foregrounds susceptibility to stressors, whereas intrinsic capacity identifies the physical and mental resources available to meet them [43]. Functional ability reflects how these resources are expressed through interaction with the living environment [23, 24]. Together, these constructs explain why older adults with apparently similar clinical profiles may respond differently to the same thermal challenge. They remain predominantly centred on the individual, however, and do not fully establish whether the personal and contextual resources, required for protection, can be mobilised during extreme heat.

## Adaptive capacity: Integrating climate science and geriatric medicine

In climate science, adaptive capacity describes the ability of individuals, communities or systems to adjust to hazards, moderate harm, cope with consequences and maintain essential functions [5, 26, 27]. It shapes how biological, social and environmental vulnerabilities translate into health outcomes.

Its integration into geriatric models of heat vulnerability remains limited. Biological susceptibility, frailty and intrinsic capacity principally characterise the individual, whereas adaptive capacity captures the dynamic interaction with the physical, social and institutional context [26, 27]. Unlike intrinsic capacity, which describes individual resources, adaptive capacity asks whether those resources can be converted into protection under actual environmental and social conditions. It is therefore a dynamic, context-dependent property that describes the ability to respond effectively under prevailing conditions and is conceptually distinct from the broader capacity to recover from stress.

This interpretation aligns with WHO Healthy Ageing, in which functional ability emerges from interaction between intrinsic capacity and the environment [23, 24]. During extreme heat, adaptive capacity reflects whether this interaction allows the individual to take effective protective action. Because it depends on both personal and contextual resources, it may change as health, caregiving, housing, energy affordability and service availability change.

In older adults, adaptive capacity therefore emerges from the continuous interaction between multiple interdependent domains. Physiological reserve determines the biological response to thermal stress; frailty and multimorbidity influence the probability of acute clinical decompensation; intrinsic capacity reflects the physical and mental resources available to the individual; while functional ability determines whether these resources can be translated into effective adaptive behaviours, including maintaining adequate hydration, modifying daily activities, operating cooling devices, reaching cooler environments or seeking timely assistance. At the same time, housing characteristics, access to affordable energy, social networks, neighbourhood resources and the organisation of health and social-care services determine whether these individual capacities can be effectively mobilised during prolonged heat exposure [5, 24, 26, 44, 45].

Importantly, these domains should not be viewed as independent or additive components, but as elements of a highly interconnected adaptive system. Older adults with comparable levels of physiological reserve and intrinsic capacity may exhibit markedly different adaptive capacity according to their living conditions. For example, adequate family support may compensate for moderate functional limitations by facilitating hydration, recognising early symptoms of heat-related illness or ensuring timely access to cooler environments. Conversely, social isolation, inadequate housing or energy insecurity may substantially reduce adaptive capacity by limiting access to even the most basic protective measures, thereby amplifying the clinical consequences of relatively modest biological vulnerability. Emerging evidence increasingly supports the importance of these interactions in determining resilience to climate-related health risks among ageing populations [44–46].

Extreme heat thus acts as a stress test of physiological reserve, clinical resilience, functional ability and environmental resources, helping to explain heterogeneous outcomes among similarly exposed older people [26, 27, 44, 45].

Comprehensive Geriatric Assessment (CGA) already assesses several heat-relevant domains, including hydration, medication exposure, cognition, mobility, function and living conditions. Heat-specific interpretation could determine whether a person can recognise risk, maintain hydration, modify activities and seek help. Additional questions should address actual indoor heat, affordable cooling, ability to use cooling devices or reach a cooler setting, and a reliable monitoring and escalation plan.

We therefore propose a small heat-specific extension to routine CGA (Table 2). Identified barriers could prompt personalised hydration or medication plans, caregiver monitoring, environmental or energy support, or access to a cooler setting. Geriatric or primary care teams, with nursing, pharmacy and social-care input, could assess these domains before the hot season and review them during alerts, treatment changes and care transitions. This author-developed proposal has not been validated; its feasibility, predictive value and clinical effectiveness require prospective evaluation.

**Table 2:** Proposed heat-specific extension of comprehensive geriatric assessment.

Domain	Information typically available from routine CGA	Heat-specific assessment to add	Action triggers and personalised responses
Clinical reserve, hydration and medicines	Frailty, multimorbidity, hydration status, renal function and medication review	Previous heat-related illness; recent heat-associated deterioration; ability to increase fluid intake safely; medicines that may impair heat dissipation, fluid balance, blood pressure, alertness or renal function; availability of an individual medication and hydration plan; appropriate medicine storage	Previous heat-related decompensation, reduced intake, postural symptoms, renal or electrolyte deterioration, or heat-risk medicines or combinations should prompt an individualised hydration and monitoring plan, medication review and clear instructions on when to seek clinical advice
Cognition and heat-risk awareness	Cognitive function, sensory impairment and capacity for self-management	Ability to perceive thirst and thermal discomfort, understand heat alerts, remember protective advice, operate cooling devices and request assistance	Inability to recognise risk or implement instructions should prompt caregiver involvement, simplified written or visual advice and scheduled monitoring during heat alerts
Mobility and functional status	Gait, balance, falls, ADL and IADL	Ability to obtain fluids, manage windows or shading, use cooling equipment, reach the coolest room, leave an overheated home and access a cooling centre safely	Dependence in heat-relevant activities should prompt practical assistance, relocation of fluids and cooling within safe reach, mobility support and an accessible plan for transfer to a cooler environment
Housing and indoor thermal exposure	Living arrangement and general home conditions	Indoor temperature, including night-time temperature where feasible; ventilation, shading, solar exposure, floor level and presence of a cooler room	Persistent indoor overheating, inadequate night-time cooling or absence of a safe room should prompt a home cooling plan, environmental modifications, equipment provision or temporary access to a cooler setting
Cooling and energy security	Financial and housing difficulties may be identified during social assessment	Availability and functioning of available and affordable cooling; ability to operate them; affordability of use; energy arrears or disconnection risk; avoidance of cooling because of cost; accessibility of public cooling spaces	Unavailable, non-functioning or unaffordable cooling should prompt equipment support, connection with energy or financial assistance, and identification of an accessible alternative cooling location
Social support and access to services	Living alone, caregiver availability, social network and formal care	Named contact responsible during heat alerts; expected frequency of calls or visits; caregiver capacity; telephone access; registration for local alerts; transport and escalation arrangements	Absence of reliable monitoring or inability to obtain help should prompt scheduled contacts or visits, caregiver education, involvement of community or social services and a documented escalation plan

*Note*: This non-validated conceptual proposal was developed by the authors by mapping routine CGA components to the heat-related risks identified in the narrative synthesis. Routine domains should be assessed using locally validated frailty, cognitive, ADL/IADL and mobility measures; heat-specific items through targeted questions and, where feasible, indoor temperature monitoring. Action triggers are illustrative; established clinical definitions and local heat-health thresholds should apply. Source: Authors’ own elaboration. ADL, activities of daily living; CGA, Comprehensive Geriatric Assessment; IADL, instrumental activities of daily living.

## Energy insecurity and the emergence of summer cooling poverty

Energy insecurity, the inability to meet household energy needs reliably and affordably, makes maintenance of safe indoor temperatures contingent on both energy access and cost [16, 47]. The European energy-price shock illustrated how geopolitical events can rapidly worsen household energy poverty [17, 48]. Evidence on its health effects has focused mainly on inadequate winter heating; its extension to residential cooling during increasingly hot summers remains less developed despite the growing need for electrically powered protection [16, 20, 47, 48].

A Joint Research Centre analysis reported that 26% of EU households could not keep their homes comfortably cool in summer, rising to almost 35% among low-income households [20, 48].

For older adults with multimorbidity, frailty or disability, inadequate cooling may increase cardiovascular strain, dehydration, sleep disruption and acute decompensation [18, 19, 49]. Summer cooling poverty arises from the interaction of energy prices and income with housing quality, building efficiency, local climate, urban form and health status. It cannot therefore be inferred from income or air-conditioning ownership alone: equipment may be unavailable, inefficient, unaffordable to operate or unusable because of cognitive or functional limitations. Older adults may also be unable to reach alternatives outside the home because of impaired mobility, cognitive impairment or limited transport. Access to safe and affordable cooling should form part of healthy-ageing and climate-adaptation strategies, while avoiding policies that rely exclusively on domestic air conditioning [16, 18–20, 47–49].

## Social frailty: Translating adaptive capacity into clinical resilience

Social frailty describes a broader loss of social resources, participation, roles and reciprocal support than social isolation or loneliness alone [50, 51]. It has been associated with disability, physical and cognitive decline, hospitalisation and mortality, even after adjustment for selected clinical and functional covariates [50–54]. In the context of extreme heat, it provides a potential link between environmental conditions and clinical vulnerability by indicating whether the social resources required to maintain autonomy and respond to stressors remain available.

Population ageing, smaller households, changing family structures, socioeconomic inequality and reduced energy affordability are altering the context in which older adults experience heat [7, 47, 55, 56]. These pressures extend beyond Europe: US evidence links disparities in heat-related mortality to unequal access to air conditioning, while studies from Singapore and Japan associate social frailty with adverse functional outcomes [49, 52–54]. The availability and meaning of family support, community participation and formal care differ across cultural and geographical settings; evidence derived mainly from high-income countries should not be assumed to represent older populations elsewhere.

Hydration, recognition of symptoms, activity modification, access to cooler environments and timely care often depend on relatives, caregivers, neighbours or community services. Erosion of these resources may reduce adaptive capacity even when intrinsic capacity is relatively preserved [50–56].

Persistent social isolation may also become biologically embedded through neuroendocrine, immune and inflammatory dysregulation, reducing physiological resilience [55–59]. These mechanisms have not been tested specifically during heat exposure but provide a plausible link between social disadvantage and reduced tolerance to thermal stress.

These behavioural and biological pathways are plausible but have rarely been examined together during heatwaves; social frailty should therefore be treated as an emerging determinant of heat-related risk rather than a validated heat-specific predictor.

Strengthening adaptive capacity requires heat resilience to be embedded across health and social-care systems. Before the hot season, services could use clinical and social data to identify high-risk older people, including those with social frailty, assign responsibility for outreach, coordinate heat-specific care plans and ensure continuity during alerts. Heat-health alert and response systems should link meteorological warnings with primary care, emergency services, hospitals, long-term care, social services, municipalities and community organisations. In rural British Columbia, locally tailored cross-sectoral planning using existing community assets facilitated implementation [60]. Preparedness remains uneven across hospitals and nursing homes, highlighting the need for heat-specific protocols, indoor temperature monitoring, reliable cooling, staff training and continuity plans, alongside systematic climate-hazard assessment of healthcare infrastructure [13, 61]. Community-based interventions may improve heat literacy and reduce heat-related symptoms, although evidence for sustained behavioural change and health outcomes remains mixed [62]. Economic evaluation should be incorporated because evidence on the costs and health benefits of local climate action remains limited and methodologically heterogeneous [63]. These measures require coordinated but distinct healthcare, social-care, energy, housing and urban-planning interventions; reliance on domestic air conditioning alone may increase household costs, emissions and inequalities.

## Discussion and limitations

Geriatric models explain vulnerability through frailty and describe how intrinsic capacity interacts with the environment to shape functional ability, while resilience concerns the response to and recovery from stress. Climate vulnerability models organise risk around exposure, sensitivity and capacity to adapt [5, 23–27]. Our perspective connects these traditions by using adaptive capacity to examine whether personal and contextual resources can be mobilised into effective protection during extreme heat. Adaptive capacity may moderate heat-related risk when protective resources alter the effect of exposure on health outcomes, while adaptive behaviours may mediate the relationship between contextual resources and those outcomes. These propositions require prospective cohorts and other longitudinal designs incorporating indoor temperature and multilevel data, followed by interventions assessing both clinical and distributional outcomes.

This review has several limitations. Evidence was selected through a structured but non-systematic process and remains concentrated in Europe and other high-income settings. Few studies have examined biological, clinical, functional, social and environmental domains simultaneously, and evidence concerning cooling poverty and social frailty during extreme heat remains partly indirect. Neither the proposed perspective nor its heat-specific extension of comprehensive geriatric assessment has been validated. These limitations constrain causal interpretation, geographical transferability and the definition of universal thresholds for action.

## Conclusions

Extreme heat is an increasing challenge for healthy ageing. Effective prevention should consider frailty, cognition, functional status and medication-related risk together with indoor heat exposure, access to affordable cooling and reliable social support. Adaptive capacity integrates these dimensions by identifying whether an older person can mobilise effective protection during a heatwave, although its proposed translation into comprehensive geriatric assessment remains conceptual.
